# Supplementary material for: A Questionnaire Survey on Long-Term Outcomes in Cats Breed-Screened for Feline Cardiomyopathy
Source: Animals (Basel). 2022 Oct 15;12(20):2782. doi: 10.3390/ani12202782 (PMC9597769; doi:10.3390/ani12202782)
Supplement: Supplementary file 1 [file animals-12-02782-s001.zip › animals-1958419-supplementary.pdf]

APPENDIX 1: THE QUESTIONNAIRE

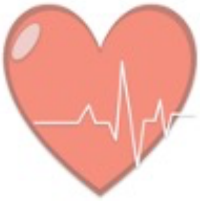

Questionnaire

Heart screening of your cat - what happened after that?

<u>All information about you and your cat will be treated with confidentiality!</u>

Mandatory answer to questions marked with *

Section A: Information about the cat and the owner

**Information about the owner**		
**First name:**	**Surname:**	**Country:**
**Street address:**	**Postal code:**	**City:**
**Email:**	**Phone number:**<br>Home:<br>Mobile:<br>Work:	

**Information about the cat**	
**Reg. no.:**	**Breed: ***
**Registered name: ***	**ID-no.: chip number and/or tattoo:**
**The cats given name:**	**Date of birth: ***

**Sex**	**Castrated ***	**Is the cat born in Sweden or imported? If it is imported, please enter the country,**
□ Male<br>□ Female	□ Yes, before/at screening occasion; date:____<br>□ Yes, after the screening occasion; date:_____<br>□ No	□ Born in Sweden<br>□ Import:____________________

Has the cat been used for breeding? *

□ Yes □ No □ Don't know If yes, how many litters/offspring did the cat get? __________________

Does the cat have any relatives who have been diagnosed with hypertrophic or restrictive cardiomyopathy? *

(Parents, littermates or offspring are counted as relatives in this study)

□ Yes □ No □ Don't know If yes, do you remember which? ________________________________

If you have previously completed the survey for another cat and thus filled in your owner information, it is enough to write that cat's ID number under the box for first name. This is so that you do not have to re-enter all of the information. For the cat's identity, it is enough to enter either ID number/tattoo or reg. no.

If the cat is DECEASED or MISSING, please continue with part 2.
If the cat is still ALIVE, please continue with part 3.
If the cat has a NEW OWNER/FOSTER HOME, please continue with part 4.

Section B: The cat is deceased or missing,

The cat's weight as an adult (c.)______ Kg
Normal body condition score (BCS) □ Under normal BCS □ Over normal BCS □

1. Is the cat *
Missing………………………………….. □
Deceased……………………………..... □

2. If missing, *
How did the cat disappear (for example; ran away, stolen, do not know, etcetera)? ____________
When did the cat disappear? ____

If missing, please continue with question 5.

3. If the cat is deceased, how did the cat die? *
Euthanasia………………………………….. □
Natural death……….……………………..... □
Other……………………………………...... □
Of other, please specify: ____

Date of death/euthanasia: * ____________________

4a. Were clinical signs detected before the cat died? *
□ Yes, from heart disease □ Yes, from another disease □ No □ Don't know
If yes, please make a note in the relevant boxes below.

4b. Clinical signs
Murmur (according to vet exam)…………...................... □
Dysrhythmia/arrhythmias (according to vet exam)…...... □
Lethargy ………...........................……….................. □
Affected breathing pattern ………..........………………… □
If affected breathing, please describe the breathing pattern: ________________________
Thrombus …………………………............................... □
Increased abdominal girth ………..........………………… □
Inappetence ………................................…………...... □
Weight loss ………...................................…………..... □
Other ………..……………… □
If other, please specify: ____

5a. Was the cat diagnosed with a heart disease before it died/disappeared/was euthanized? *
□ Yes, HCM/RCM □ Yes, HCM/RCM and other heart disease □ Yes, other heart disease
□ No □ Don't know
If yes, date of diagnosis: ____

If No, please continue with question 10.

5b. How was the heart disease in the previous question diagnosed/suspected?
Ultrasound ………........................……………………..... □
X-ray …………………………………............................... □
Autopsy …………………………….................................. □
Other analysis (genomic test etcetera)………….……........... □
Don't know ……………………………............................ □
Other ………………………………………………………… □
If other, please specify: ____

6. Did the cat show any clinical signs and receive treatment for the heart disease? *

A. HCM/RCM, untreated, no clinical signs (other than murmur) from the heart………… □
B. HCM/RCM, received medical treatment but never had any clinical signs from the heart…………………………………………………………………………………………….. □
C. HCM/RCM, received medical treatment and had clinical signs from the heart.......... □

6b. Treatment for the cat's heart condition *

A. Which medications were given to the cat? ____
B. Under how long was the cat treated for? ____

6c. Response to treatment *

A. No difference after treatment was initiated □
B. Small improvement after treatment was initiated □
C. Obvious improvement after treatment was initiated □
D. Worsening after treatment was initiated □

7. If the cat was diagnosed with HCM or RCM, did the veterinarian assess that your cat developed congestive heart failure (respiratory distress, fluid in the lungs and/or abdomen, etcetera) at any stage of the disease?

□ Yes □ No □ Don't know

If yes and the cat was diagnosed with HCM/RCM and developed congestive heart failure, for how long did the cat live after diagnosis with congestive heart failure?_________________________________
Which medications were given to the cat? ____
How long was the cat treated for?____

8a. Was any X-ray examination of the thorax performed during the investigation of the heart disease?

□ Yes □ No □ Don't know

8b. Findings in the X-ray examination:

Pulmonary edema/fluids in the lungs............ □
Enlarged heart …………………………....... □
Other…………………………………...…… □
If Other, please specify: ____
Don't know……………………………… □

9. If the cat was diagnosed with HCM or RCM, did the veterinarian assess that your cat developed arterial thromboembolism/thrombus with paralysis of the extremities, tail or other clinical signs at any stage of the cat's life?

□ Yes □ No □ Don't know

10. Was the cat diagnosed with any disease (non-heart related) other than the heart diseases HCM or RCM?

□ Yes □ No □ Don't know

If yes, which disease/s? ____
How was the diagnosis made?____

11. Cause of death/euthanasia

HCM (congestive heart failure) ……………...…………. □
RCM (congestive heart failure) ……………...…………. □
HCM - other than congestive heart failure, please specify____________________________________
RCM - other than congestive heart failure, please specify ____________________________________
Other heart disease …………………………………....... □
Asthma ………………………………………………….. □
Kidney disease ………………………………............... □

Urinary tract disease………………………………………. □
Tumor………………………………………………………. □
Joint disease………………………………………………. □
Trauma/accident………………………………………….. □
Infectious disease………………………………………… □
Examined, but diagnosis not established………………… □
Don't know (not examined)……………………………….. □
Other …………………………………………………….. □
If other, please specify; ____

12a. Was an autopsy performed? * □ Yes □ No □ Don't know
If yes, where was the autopsy performed (for example the SVA/SLU, or local vet?________________

12b. Postmortem diagnosis
HCM (congestive heart failure) ……………………… □
RCM (congestive heart failure) ……………………… □
HCM - other than congestive heart failure, please specify_________________________________
RCM - other than congestive heart failure, please specify _________________________________
Other heart disease …………………………………… □
Asthma ………………………………………………….. □
Kidney disease ………………………………………… □
Urinary tract disease……………………………………. □
Tumor……………………………………………………. □
Joint disease…………………………………………….. □
Trauma/accident………………………………………… □
Infectious disease……………………………………….. □
Cause of death not established…………………………. □
Don't know………………………………………………. □
Other ……………………………………………………. □
If Other, please specify; ____

If you have any information from veterinary visits (and wish to share it);
The owner gives us (J. Häggström, I. Ljungvall, Å. Ohlsson Andersson, A. Follby or A. Pettersson) permission to access the cat's medical records:
□ Yes □ No
These data are of course treated with confidentiality!

Any own comments about the questionnaire, your cat or the feline heart screening program?
____
____
____

Thank you for your participation!

The results will eventually be published on the SLU Library page (epsilon), where the master's thesis will be found under the names Anna Follby and Anna Pettersson, respectively.

Section C; The cat is alive,

The cat's weight as an adult (c.)______ Kg
Normal body condition score (BCS) □ Under normal BCS □ Over normal BCS □

1. The owner's assessment *

A. No clinical signs of heart disease (and no diagnosis with HCM/RCM)…………………… □
B. HCM/RCM, untreated, no clinical signs from the heart.. □
C. HCM/RCM, receives medical treatment but have never had any clinical signs from the heart………………………………………………………………………………..... □
D. HCM/RCM, receives medical treatment and had clinical signs from the heart.................. □

2. If you answered A on question 1, that is "I No clinical signs of heart disease (and no diagnosis with HCM/RCM)", has a veterinarian been listening to the heart after the last screening?
Skip this question and the next one if you have answered B, C or D on the previous question.

□ Yes □ No □ Don't know

If you answered A on question 1 and yes on question 2: did the vet hear any murmur?

□ Yes □ No □ Don't know

If you answered D, continue with the remaining questions, if you answered C, please go to question 6, if you answered B, please go to question 8, if you answered A, please go to question 10.

3. If your cat has been diagnosed with hypertrophic or restrictive cardiomyopathy, did the veterinarian assess that your cat developed congestive heart failure (respiratory distress, fluid in the lungs and/or abdomen, etcetera) at any stage of the disease? *

□ Yes □ No □ Don't know

Which medications is the cat being treated with? ____
How long has the cat been treated for? ____
Starting date for the medications:____
If the medication has been stopped, date:__________________________________
Voluntary comment about the medication:____

4. If the cat was diagnosed with HCM or RCM, did the veterinarian assess that your cat developed arterial thromboembolism/thrombus with paralysis of the extremities, tail or other clinical signs at any stage of the disease? *

□ Yes □ No □ Don't know

5. Clinical signs *

Murmur (according to vet exam)………….................... □
Dysrhythmia/arrhythmias (according to vet exam)……. □
Lethargy ………......................………................... □
Affected breathing pattern ……….........…………… □
If affected breathing, please describe the breathing pattern: _________________________
Thrombus ……………………………….................... □
Increased abdominal girth ……….........…………… □
Inappetence ……….....................…………...... □
Weight loss ……….......................…………..... □
Other ……….......................................……… □
If other, please specify: ____

6. The cat has never shown any clinical signs from the heart. Which treatment has been used?

Medication*: ____
Starting date: ____
If terminated, ending date: ____

7. Response to treatment *

A. No difference after treatment was initiated □
B. Small improvement after treatment was initiated □
C. Obvious improvement after treatment was initiated □
D. Worsening after treatment was initiated □

8a. Was an additional ultrasound examination of the heart performed during the investigation of the heart disease or any other disease (besides heart screening)? *

□ Yes □ No □ Don't know

8b. Diagnosis at the ultrasound examination?

Hypertrophic cardiomyopathy.. □
Restrictive cardiomyopathy.. □
Congenital heart defect .... □
Other heart disease ……………....................................... □
No findings…………………………………………... □
Other.. □

If Other heart disease or Other, please specify: ______________________________________

9a. Was any X-ray examination of the thorax performed during the investigation of the heart disease or other disease?*

□ Yes □ No □ Don't know

9b. Findings in the X-ray examination:

Pulmonary edema/fluids in the lungs............ □
Enlarged heart …………………………....... □
Other………………………………………...… □

If Other, please specify: ____

Don't know………………………………… □

10. Has the cat been diagnosed with any other disease than the heart disease HCM/RCM?*

□ Yes □ No □ Don't know

If yes, which? ____

How was the diagnosis confirmed?____

The owner gives us (J. Häggström, I. Ljungvall, Å. Ohlsson Andersson, A. Follby or A. Pettersson) permission to access the cat's medical records:

□ Yes □ No

These data are of course treated with confidentiality!

Any own comments about the questionnaire, your cat or the feline heart screening program?

____

____

____

Thank you for your participation!

The results will eventually be published on the SLU Library page (epsilon), where the master's thesis will be found under the names Anna Follby and Anna Pettersson, respectively.

Section D; The cat has a new owner/foster home,

Please share the contact information for this person, so that we are able to follow up the cat.

**Information about the new owner/foster home**		
**First name:**	**Surname:**	**Country:**
**Street Address:**	**Postal number:**	**City:**
**Email:**	**Phone number:**<br>Home:<br>Mobile:<br>Work:	

Any own comments about the questionnaire, your cat or the feline heart screening program?

____

____

____

Thank you for your participation!

The results will eventually be published on the SLU Library page (epsilon), where the master's thesis will be found under the names Anna Follby and Anna Pettersson, respectively.
